# Skin Color and Self-reported Sun Exposure Scores are Associated with Serum 25-Hydroxyvitamin D Concentrations in a Multi-ethnic Population Living in South Florida

**DOI:** 10.9734/bjmmr/2014/10551

**Published:** 2014-07-25

**Authors:** Sahar Ajabshir, Joel C. Exebio, Gustavo G. Zarini, Ali Nayer, Michael McLean, Lemia Shaban, Fatma G. Huffman

**Affiliations:** 1 Department of Dietetics and Nutrition, Robert Stempel College of Public Health and Social Work, Florida International University, Miami, USA.; 2 Division of Nephrology and Hypertension, Miller School of Medicine, University of Miami, Miami, USA.; 3 Department of Food Science and Nutrition, College for Life Sciences, Kuwait University, Kuwait City, Kuwait.

**Keywords:** Serum vitamin D, vitamin D deficiency, UV exposure, vitamin D intake

## Abstract

**Aims:**

The aim was to investigate the association between serum 25-hydroxyvitamin D [25(OH)D], skin color and sun exposure score.

**Study Design:**

Cross-sectional.

**Place and Duration of Study:**

Florida International University, Robert Stempel College of Public Health and Social Work, Department of Dietetics and Nutrition, Miami, Florida from July 2012 to October 2012.

**Methodology:**

Seventy six adults, ages 18–36 years living in South Florida participated in the study. Skin color was quantified by a IMS Smart Probe 400 scanner and 25(OH)D was measured by ELISA. A sun exposure questionnaire was used to record the weekly sun exposure scores. A food frequency questionnaire was used to record daily vitamin D intake.

**Results:**

Multiple-linear regression analysis indicated that sun exposure, forearm skin color and vitamin D intake were significant predictors of 25(OH)D (*P*=.004, *P*=.003 and *P*=.021 respectively). This association held after controlling for covariates (B=.371, *P*=.027 for forearm, B=.031, *P*=.005 for total sun exposure and B=.689, *P*=.003 for vitamin D intake).

**Conclusion:**

Skin color, sun exposure along with vitamin D intake may be used as an indirect non-invasive tools to estimate 25(OH)D levels in healthy individuals in South Florida.

## 1. INTRODUCTION

Hypovitaminosis D is associated with a wide range of health concerns, such as osteomalacia, diabetes, cardiovascular complications, autoimmune conditions and certain cancers [1–7]. While vitamin D can be obtained through dietary sources [8–9], the vitally important level of bodily vitamin D is provided by its endogenous ultraviolet–dependent (UV) biosynthesis, through photochemical conversion of 7-dehydrocholesterol in the skin [7]. Hepatocytes convert vitamin D to 25-hydroxyvitamin D [25(OH)D] [10]. Circulating 25(OH)D concentration is widely used as the most acceptable indicator of vitamin D status due to its half-life which is indicative of exposure to sun within two months [9,11,12]. Environmental conditions such as geographical location, latitude, seasons and atmosphere along with personal characteristics such as age, gender, race, skin color, sun protective behaviors, dietary intakes and body mass index (BMI) can potentially affect the serum 25(OH)D level [13–26].

Previous studies using sun exposure questionnaires to predict serum vitamin D levels have shown significant results [27–31]. However, the correlations have been in the range of .29–.39, leaving a large percentage of the variation in serum vitamin D unexplained by sun exposure alone. This indicates that other factors such as age and skin color should be included in the model in order to increase the predictive power. Hanwell et al. [32] showed that the correlation between serum vitamin D and sun exposure is only significant for the summer months, but not for winter in hospital workers in Italy, meaning that even when workers spent several hours under the sun in winter it has no effect on serum vitamin D levels and other factors like vitamin D intake maybe more relevant during these months [32]. Therefore, using self-reported sun exposure alone leads to a high probability of misclassification of vitamin D status. Another variation in estimating vitamin D status could be ethnicity. Our previous study showed significant variation in serum vitamin D levels across ethnicity among individuals residing in South Florida [33].

Measuring serum 25(OH)D level requires an invasive and expensive process. Developing validated noninvasive methodology to predict vitamin D levels are needed. Methods that can easily be used in a clinical setting without training could facilitate screening for hypovitaminosis D. Providing a new methodology to clinicians to predict vitamin D deficiencies quickly will lead to prevention of vitamin D deficiency and related complications. Therefore, the aim of this study was to develop non-invasive and inexpensive methodology to predict serum 25(OH)D among a multi-ethnic population living in a subtropical climate, Miami, Florida.

## 2. MATERIALS AND METHODS

This study was approved by Institutional Review Board (IRB) and all participants signed an informed consent form.

### 2.1 Participants

In this cross-sectional study participants were recruited from the Florida International University communities by flyers and word of mouth. The inclusion criteria was to be older than 18 year, not taking vitamin D supplements, living in South Florida for more than one year and not majoring in the nutrition field. A sample of 85 participants aged between 18 and 36 years were enrolled. The study was conducted during the months of July 2012 to October 2012. The average daily temperature for the month of June was 82.6°F with an average rainfall of 12.56 inches in Miami [34]. Similarly, the average daily temperature was 83.4°F with an average rainfall of 8.92 inches and 83.8°F with 15.92 inches for the months of July and August, respectively.

### 2.2 Skin Color

In order to have an objective measure of sun exposure, skin color was determined by reflectance colorimetry using the IMS Smart Probe 400, Millford, CT, USA. This instrument uses the International Commission on Illumination Scale which ranges from 0 (black) to 100 (white) for skin color. Two readings at each measurement site (6 readings total) for each participant were taken: Two on the wrist of the right hand (area most exposed to sun), two on the inside of the right upper arm and the waist (areas less exposed to the sun). The mean values were used. The change in skin color due to sun exposure was calculated by finding the difference between the less exposed area (natural color) and the most exposed area.

### 2.3 Blood Collection

Fasted (8–10 hours) venous blood (10ml) was collected from each subject by a certified phlebotomist using standard laboratory methods. Serum vitamin D concentrations were measured with an enzyme-immunoassay kit by absorbance (Immunodiagnostic Systems Scottsdale, AZ, USA).

#### 2.3.1 Demographics questionnaire

A self-administered socio-demographic questionnaire was used to collect data on age, education, race/ethnicity, employment status, health insurance, alcohol use, marital status and smoking status.

### 2.4 Anthropometrics

The participants were asked to step behind a screened area, remove their shoes and heavy clothing and step on a scale with height rod to measure their body weight and height. The participants’ waist and hip circumferences were measured with a non-flexible tape measure.

### 2.5 Sun Exposure Questionnaire (SEQ)

The instrument developed by Hanwell et al. [32] was applied following the original rubric. Time spent outdoors during the previous week (0<5 minutes, 1=5–30 minutes and 2=>30 min) was self-reported. Four options for skin exposed while outdoors were offered (1=face and hands, 2=face, hands and arms, 3=face, hands and legs and 4=bathing suit). The daily sun exposure score for each day was calculated by multiplying the time spent outdoors score times the skin exposed while outdoors score. The scale for each day ranged from 0 to 8. The weekly sun exposure was calculated by adding the daily scores (min=0, max=56).

### 2.6 Short Vitamin D and Calcium Questionnaire (FFQ-D)

The short instrument for assessing dietary intakes of calcium and vitamin D by Blalock and colleagues was used [35]. The original instrument included 22 foods and beverages. The new calculator has added pizza for a total of 23 foods. The questionnaire asked for the frequency of consumption of each food and the serving size.

### 2.7 Statistical Analysis

The descriptive statistics for continues variables was presented as mean ± SD and proportions for categorical variables. Spearman’s correlation among weekly sun exposure scores, delta of skin color, sun exposure scores with serum vitamin D and short food frequency questionnaire with serum vitamin D were run. The relationship between serum 25(OH)D, sun exposure and skin color was evaluated by a multi-linear regression models. Serum 25(OH)D was the dependent variable. Total sun exposure score and forearm skin color were independent variables. The three variables were all continuous. A simple model was run using total sun exposure and forearm skin color as predictors of serum 25(OH)D. Confounding factors including daily vitamin D intake, age, gender, BMI, years living in US, race, tobacco use and alcohol consumption were added to the adjusted model. Two-way and three-way interactions were tested between total sun exposure score, forearm skin color, daily vitamin D intake, and gender. Analyses were conducted using SPSS version 19 (Chicago, IL, US).

## 3. RESULTS AND DISCUSSION

This cross-sectional study included 85 participants. Nine participants were excluded; 4 subjects due to refusing blood draw and 5 subjects due to missing values for sun exposure questionnaire. Final sample considered for the analysis included 76 participants, males (n=39), females (n=37). Descriptive characteristics are provided in Table 1. The mean age was 25.32±4.82 years. Forty-eight percent were females and the mean BMI was 24.49±4.10 kg/m^2^. The mean serum 25(OH)D, forearm skin color and total sun exposure were 24.53±9.65, 56.17±7.35 and 28.05±12.19, respectively. The minimum score for weekly sun exposure was 14 and the maximum 40. About sixty percent of the participants were White and about forty percent were Blacks and Asians. Vitamin D insufficiency (25(OH)D <30 ng/ml) was present in 77.6% of the participants.

**Fig. 1.**
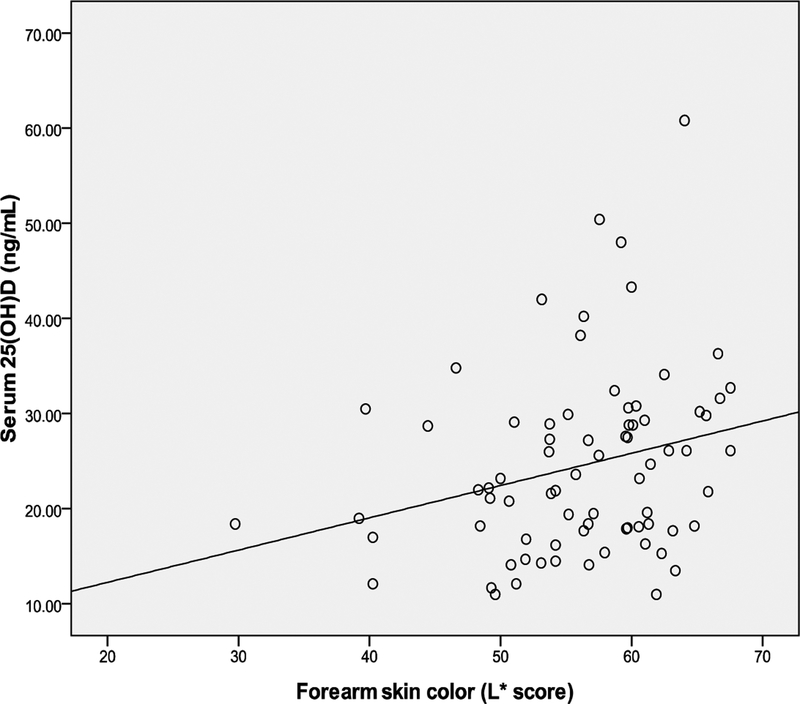
Correlation between serum 25(OH)D and forearm skin color

**Fig. 2.**
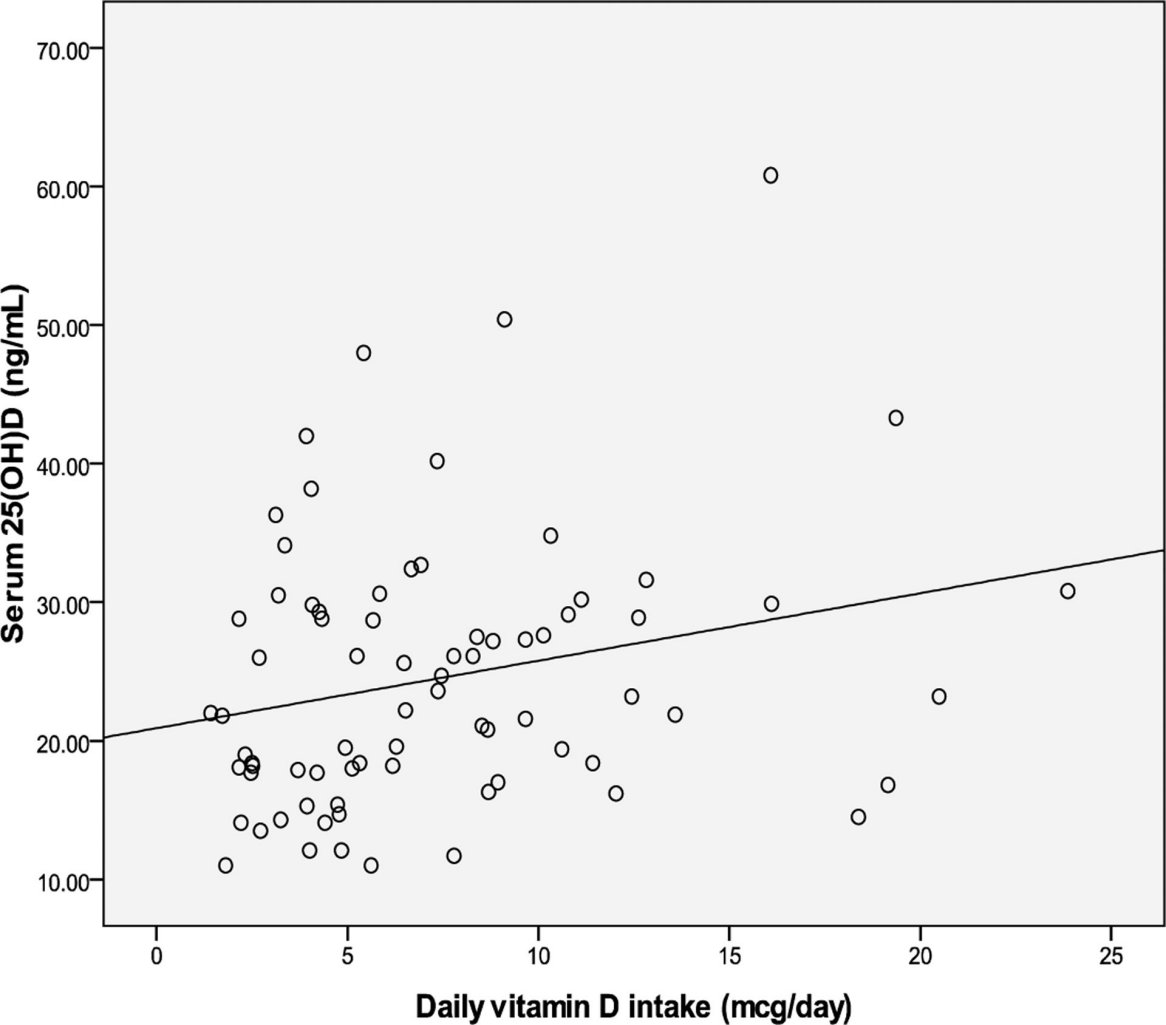
Correlation between serum 25(OH)D and vitamin D intake

Correlations between weekly sun exposure and change in skin color, weekly sun exposure and serum vitamin D, and vitamin D intake and serum vitamin D were significant (*P*=.037, r=.24 and *P*=.05, r=.23 and *P*=.03 and r=.25 respectively) (Table 2 and Figs.1, 2, and3).

The unadjusted model showed that total sun exposure (*P*=.004), forearm skin color (*P*=.003) and daily vitamin D intake (*P*=.021) were good predictors of serum 25(OH)D. In this model, 22 percent of the variation in serum 25(OH)D was explained by total sun exposure, skin color and daily vitamin D intake. This relationship remained significant (*P*=.005 for sun exposure, *P*=.027 forearm skin color and daily vitamin D intake *P*=.003) after controlling for covariates, including age, gender, BMI, years living in US, race, tobacco use and alcohol consumption. The fully adjusted model explained 34.5 percent of the variation in serum 25(OH)D. For every one unit increase in forearm skin color score, total sun exposure and daily vitamin D intake, there was a .37, .03 and .69 unit increase in 25(OH)D, respectively, keeping the other variables constant (Table 3). Two and three-way interactions between daily vitamin D intake, total sun exposure score, forearm skin color and gender were not significant.

This study examined the association between forearm skin color, sun exposure score and daily vitamin D intake with serum 25(OH)D concentration. The results showed forearm skin color, sun exposure score and daily vitamin D intake predicted serum 25(OH)D among our participants. These findings suggest that people with lighter skin color have higher concentrations of serum 25(OH)D. Our results are consistent with similar studies that reported darker skin color is associated with serum 25(OH)D insufficiencies or deficiencies [36–38]. We also measured the unexposed skin color in two areas and calculated the delta between exposed and un-exposed areas; however, only exposed (forearm) skin color was significantly associated with serum 25(OH)D concentrations. The correlations between delta of exposed and un-exposed areas, sun exposure and serum vitamin D were not significant. This is in contrast to the findings of Nessvi et al. [15] who suggested that serum 25(OH)D is correlated with both constitutive (unexposed) and facultative (exposed) skin color. The reason for this difference may be because their study was done during non-summer seasons, in which people may not have significant exposure to sun. This can result in a non-significant difference between the exposed and exposed areas skin color. Our study was conducted during summer. Also, the exposed areas in that study were forehead and outer arm, while we considered forearm skin color as our sun exposed area. However, in non-summer seasons, the outer arm may be covered and it may not reflect the most sun exposed skin area. There are several studies confirming that forearm and inner upper arm skin colors are valid measures of exposed and unexposed areas [17,22,39,40]. Tanning behaviors can also affect the color of unexposed skin area and changes the delta between constitutive and facultative skin colors. Although the ratio between upper arm skin color and forearm skin color had a stronger correlation with serum 25(OH)D in comparison to the ratio between stomach and forearm skin color with serum vitamin D; none of these correlations were statistically significant.

The sun exposure questionnaire used in this study has not been validated for South Florida. Most studies validate sun exposure questionnaires against observed records of sun exposure or dosimetry [41–45]. However, these methods are not considered as a gold standard. Others use serum vitamin D as the gold standard due to its ability to reflect recent sun exposure [32]. In our study, weekly sun exposure scores were significantly correlated with change in skin color due to sun exposure and serum vitamin D. However, the correlations were weak (0.226 and 0.240), leaving a large proportion of variation in serum vitamin D unexplained. The fully adjusted model including sun exposure score, vitamin D intake and forearm skin color as predictors, explained 34.5% of the variation in serum vitamin D meaning that still 65% is not explained.

Other factors like the use of sunscreen, umbrellas, or days outside South Florida were not investigated and may have played a role in serum vitamin D prediction. Previous studies have shown the use of sunscreen with a SPF of 8 completely suppressed the cutaneous synthesis of vitamin D [24,46]. In addition, the vitamin D food frequency questionnaire did not include ethnic foods that may have contributed to the serum vitamin D levels like sardines and mixed ethnic dishes with cheese. The use of multivitamins containing vitamin D and the intake of vitamin D fortified foods, such as orange juice were not measured.

It is important to note that despite the young age of our sample and the majority being Caucasian, over three-quarters of the participants (77.6%) were vitamin D insufficient. It is almost paradoxical that in a tropical area like South Florida and during the summer months, the incident of vitamin D insufficiency is still high. Mean sun exposure scores were in the middle of the scale, reflecting not much outdoor activity. Similarly, the mean vitamin D intake was 7.43 mcg/day. The recommended dietary allowance for vitamin D between the ages of 19–50 years old is 15 mcg/day [47]. Alcohol intake can also interfere with vitamin D absorption and activation in the liver. In our sample, 42.1% of the subjects reported drinking one or more alcohol servings per week (12 oz of beer, 5 oz of wine or 1 oz hard liquor). The low vitamin D intake, limited sun exposure and alcohol consumption may explain in part the high incidence of insufficiency in our sample.

We suggested that higher sun exposure scores resulted in greater concentrations of 25(OH)D. These findings are consistent with Malvy et al. [48] who reported serum 25(OH)D concentration was associated with the results of self-reported sun exposure among 1191 French adults. Kimlin et al. [18] reported that regular winter sunscreen user who are involved in outdoor activities had some of the highest levels of 25(OH)D concentrations among adults living in New Zealand; however, we could not find any significant correlations between sun screen application and variance in 25(OH)D level. This may be due to our smaller group of participants and different climates. Limitations of our study included small sample size, limited age range (18–36 years old), self-reported questionnaires, cross-sectional study design, and lack of access to other regions in South Florida. Since approximately 60% of our participants were Whites and 40% were Blacks and Asians, we did not have enough power to categorize our participants into ethnic groups and assess the sun exposure behaviors and vitamin D intake behaviors among them. Another limitation of our study was not considering hepatic function. After transportation of vitamin D to the liver by vitamin D binding protein, vitamin D-25-dehydroxylase converts it to 25(OH)D and the function of this enzyme can affect the amount of circulating 25(OH)D [49,50].

## 4. CONCLUSION

Forearm skin color, sun exposure scores along with daily vitamin D intake were indirect noninvasive tools to estimate serum 25(OH)D level in young healthy individuals living in South Florida. Sun exposure scores were significantly correlated with exposed (forearm) skin color and serum vitamin D which indicates that sun exposure questionnaire is appropriate for this particular sample. Further studies should be conducted among populations with more variation of skin colors and a greater age range to confirm these results.

## Figures and Tables

**Fig. 3.**
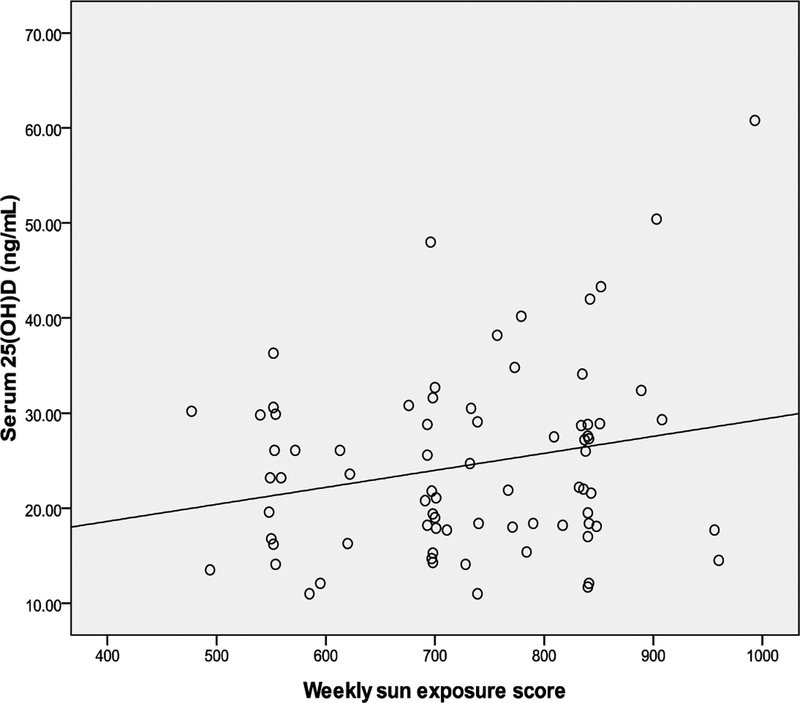
Correlation between serum 25(OH)D and sun exposure score

**Table 1.** Characteristics of participants (n = 76)

Variable	Mean ± SD
Age (years)	25.3±4.8
Gender	
_Female	48.7%
_Male	51.3%
BMI (kg/m^2^)*	24.5±4.1
Living in US (years)	10.4±9.3
Vitamin D intake (mcg/day)	7.4±4.9
Serum 25(OH)D (ng/mL)	24.5±9.7
Forearm skin color	56.2±7.6
Sun exposure score	28.1±12.2
Race	
_White	60.5%
_Black and Asian	39.5%
Tobacco use	
_No	92.1%
_Yes	7.9%
Alcohol consumption	
_No	57.9%
_Yes	42.1%

Data are % or mean **±** standard deviation (SD).

* BMI = Body mass index

**Table 2.** Correlation analysis (n = 76)

Variables compared	*r*	*P*-value
Sun exposure vs. change in skin color	.240	.04
Sun exposure vs. serum vitamin D	.226	.05
Vitamin D intake vs. serum vitamin D	.246	.03

† *P*<.05 is considered significant

**Table 3.** Relationship of serum 25(OH)D and covariates

Parameter*	*β*	*SE*	*T*	*P*-value^†^
Forearm skin color	.371	.164	2.258	.027
Weekly sun exposure score	.031	.011	2.898	.005
Daily vitamin D intake	.689	.225	3.059	.003

* Other covariates in the multiple linear regression were age, gender, BMI, years living in US, race, tobacco use and alcohol consumption;

† *P*<.05 is considered significant
